# Management of Advanced Bladder Cancer: An Update

**Published:** 2018-05-01

**Authors:** Emily A. Lemke, Amishi Yogesh Shah

**Affiliations:** Department of Genitourinary Oncology, The University of Texas MD Anderson Cancer Center, Houston, Texas

## Abstract

Bladder cancer is the sixth most common cancer in the United States; therefore, the majority of clinicians working in the oncology setting will care for this patient population. Unfortunately, treatment plans, especially in the advanced setting, lack consistency. This, along with the advanced age and comorbidities of most bladder cancer patients, can provide challenges for clinicians when developing treatment plans. In the past 2 years, new drug approvals, specifically those for immune checkpoint inhibitors, have changed the treatment landscape for bladder cancer for the first time since the 1980s. This review article outlines the current management for muscle-invasive and metastatic bladder cancer, while also highlighting future considerations in this disease space. It is imperative that oncology advanced practice providers are up to date with these new changes and have a sound understanding of treatment principles for patients with advanced bladder cancer in order to deliver the safest and most effective care.

Bladder cancer is the sixth most common malignancy in the United States, with 81,190 new cases and 17,240 deaths from bladder cancer predicted in 2018 (American Cancer Society, 2018). The most common presenting symptom is hematuria, although dysuria, frequency, and urgency occur in a certain subset of patients as well (Solomon & Hansel, 2016). The most common risk factor in the United States is smoking; other risk factors include exposure to arsenic or nitrosylating chemicals (Solomon & Hansel, 2016). The average age of diagnosis is 65, and given the high prevalence of smoking history in bladder cancer, patients often have multiple comorbidities to be considered during treatment planning (Clark et al., 2016).

Bladder cancer is categorized as either nonmuscle invasive or muscle invasive, and this differentiation is key to treatment planning and prognosis. Further classification is based on grade and histology. The majority of nonmuscle-invasive bladder cancers are managed by transurethral resection and intravesical chemotherapy and immunotherapy; chemotherapy and cystectomy have been the mainstay of muscle-invasive disease management (National Comprehensive Cancer Network [NCCN], 2018). Despite the prevalence of bladder cancer, treatment plans lack consistency, especially in the setting of muscle-invasive and metastatic disease. Furthermore, new immune checkpoint inhibitors, which have demonstrated efficacy in the metastatic setting, are opening up treatment options for the first time since the 1980s (Campbell, Siefker-Radtke, & Gao, 2016). The aim of this review article is to present the current management strategies for advanced bladder cancer.

## DIAGNOSTIC WORKUP

Hematuria is the most common symptom that initiates the diagnostic workup for urothelial carcinomas; it also has the strongest correlation to urothelial cancers (Kamat et al., 2016). Initial evaluation with cystoscopy is the first step to determine if a lesion is present; if cystoscopy yields a bladder lesion, subsequent transurethral resection of the bladder tumor (TURBT) to confirm tissue diagnosis and evaluate the extent of the disease is completed (NCCN, 2018). In the setting of muscle-invasive disease, a complete staging workup including computed tomography (CT) or magnetic resonance imaging of the abdomen and pelvis is warranted prior to initial TURBT (NCCN, 2018). In addition, a urologic exam under anesthesia (EUA) is a key part of evaluating for T3 disease. Additional testing, such as a bone scan, can be considered in the setting of an elevated alkaline phosphatase (NCCN, 2018).

## DISEASE CHARACTERISTICS

**Histology**

The majority (90%) of urothelial carcinomas are histologically transitional cell carcinoma; the remaining 10% are considered variant histologies and include squamous, small cell, sarcomatoid, micropapillary, adenocarcinoma, and plasmacytoid features (Humphrey, Moch, Cubilla, Ulbright, & Reuter, 2016; Kantarjian & Wolff, 2016). Any identified adenocarcinoma should prompt clinicians to consider a urachal tumor or metastatic disease from another primary site of disease (Kantarjian & Wolff, 2016), as these are much more likely than a true bladder adenocarcinoma. Most variant histologies are thought to portend a poor prognosis; small cell carcinoma of the bladder is an especially aggressive variant, with the potential to metastasize to the brain, as is seen in small cell cancers arising from other sites, such as the lung (Siefker-Radtke et al., 2009).

**Grade**

The 2004 World Health Organization (WHO) grading systems categorize urothelial carcinomas as either low grade, high grade, or papillary neoplasm of low malignant potential; the 2016 WHO grading systems are essentially the same as those from 2004 (Humphrey et al., 2016; Kamat et al., 2016). Papillary neoplasm of low malignant potential is a thickened urothelium with scant or no cytological atypia and no true papillary fronds; its clinical significance is not well understood (Humphrey et al., 2016).

**Stage**

Outside of small cell histology, staging is the best prognostic factor for urothelial cancer and is based on the depth of invasion as well as sites of metastatic disease (Kamat et al., 2016). Clinical staging consists of bimanual examination, cystoscopy, and complete radiologic assessment, usually consisting of a CT of the abdomen and pelvis (NCCN, 2018). Pathologic staging remains the gold standard; however, this can prove challenging, as TURBT specimens are often fragmented secondary to cautery (Kamat et al., 2016). Establishing whether the tumor is muscle invasive is key to choosing appropriate treatment. Once grade and stage are established, treatment planning and recommendations can follow. Involvement of pelvic lymph nodes is considered metastatic disease, although if a patient has significant downstaging with therapy, occasionally, surgery is still considered for a curative intent approach.

## MUSCLE-INVASIVE BLADDER CANCER

Neoadjuvant chemotherapy (NAC) followed by radical cystectomy has been established as the gold standard for muscle-invasive bladder cancer (MIBC; Grossman et al., 2003; Witjes et al., 2014). Grossman and colleagues (2003) established the role of NAC, proving significant overall survival (OS) as compared to cystectomy alone with neoadjuvant methotrexate, vinblastine, doxorubicin (Adriamycin), and cisplatin (MVAC) in MIBC. Advantages of this approach include a low burden of micrometastatic disease, as well as presumed improved tolerance prior to resection as compared to the adjuvant setting (Witjes et al., 2014). Only cisplatin-based regimens have proven beneficial in this setting (Advanced Bladder Cancer Meta-Analysis Collaboration, 2005). Neoadjuvant chemotherapy should be even more strongly considered in the setting of high-risk features, which include variant histology, palpable mass under EUA, lymphovascular invasion on pathology, tumor arising from a diverticulum, or presence of hydronephrosis (Kantarjian & Wolff, 2016). Patient comorbidities, common in this population, necessitate finesse on the part of the clinician to choose the best regimen for each individual patient.

**Neoadjuvant Therapy Regimens**

The two bread-and-butter neoadjuvant cisplatin-based regimens in bladder cancer are MVAC and gemcitabine/cisplatin (GC); the current standard is for 4 cycles total (Clark et al., 2016). Recent data suggests that using a dose-dense strategy (i.e., 2-week schedule with growth factor support) with MVAC has yielded a quicker time to surgery and improved treatment tolerance, while also demonstrating promising higher complete pathologic response rates, and thus possibly a higher chance of cure (Choueiri et al., 2014; Plimack et al., 2014). Ongoing trials continue to investigate the superiority of dose-dense MVAC (ddMVAC) vs. GC; currently, the COXEN trial, a phase II study of "coexpression with neoadjuvant chemotherapy for localized, muscle-invasive bladder cancer," is accruing nationally to answer this question (ClinicalTrials.gov, 2017). Key considerations in toxicity monitoring include kidney function, peripheral neuropathy, and hearing loss for cisplatin-based regimens; the addition of anthracycline chemotherapy in ddMVAC also necessities the monitoring of ejection fraction. Close toxicity monitoring is key during any of these chemotherapy regimens; patients are susceptible to dehydration, infection, electrolyte derangements, cytopenias, and other side effects that warrant frequent supportive care interventions.

Certain variant histologies, specifically small cell bladder cancer, require alternative neoadjuvant regimens, similar to those used in the small cell lung cancer patient population (Kantarjian & Wolff, 2016). There is some data to support the use of alternating doublet chemotherapy in this setting with ifosfamide/doxorubicin (IA) and etoposide/cisplatin (EP; Siefker-Radtke et al., 2009). Given the propensity of small cell urothelial carcinoma to metastasize to the brain, there is also a subset of patients who may benefit from prophylactic cranial irradiation (Siefker-Radtke et al., 2009).

For patients with renal insufficiency, deeming them ineligible for cisplatin-based therapy, a triplet of gemcitabine, paclitaxel (Taxol), and doxorubicin (GTA) given every 2 weeks has demonstrated clinical utility (Pagliaro, Munsell, Harris, Carolla, & Siefker-Radtke, 2011). If renal insufficiency is a result of ureteral obstruction, not uncommon in urothelial cancer, nephrostomy tubes demonstrate efficacy in decompressing the kidney, thus allowing for cisplatin regimens. Nephrostomy tubes are preferable to stents, given the tendency of stents to clot or bleed in the setting of thrombocytopenia and collapse from a growing tumor (Kantarjian & Wolff, 2016).

**Bladder Preservation**

Bladder-preserving strategies most commonly involve a combination of TURBT, chemotherapy, and radiation. Concurrent chemoradiation has proven superior to radiation alone (James et al., 2012). Therefore, most bladder preservation treatment plans include concurrent chemoradiation following TURBT, given the established role of chemotherapy as a radiosensitizer (Mak et al., 2014; Witjes et al., 2014). Cisplatin is the most commonly used; however, 5-fluorouracil (5-FU) plus mitomycin C, and gemcitabine are acceptable alternatives (Kamat et al., 2016; Witjes et al., 2014). Mak and colleagues (2014) reported a complete response in 69% of patients treated with bladder-preserving multimodality treatment in a pooled analysis of multiple prospective RTOG protocols. Patients with conventional urothelial histology, minimally invasive T2 disease, complete resection at TURBT, and without tumor-related hydronephrosis demonstrate the most robust outcomes (Kamat et al., 2016; Kantarjian & Wolff, 2016; Mak et al., 2014). Multimodality bladder preservation should be considered for patients with MIBC who are not surgical candidates, those especially motivated to keep their bladders, and those older than 75 years, who are often considered an undertreated population in evaluation for curative treatment (Kamat et al., 2016; Mak et al., 2014).

## METASTATIC BLADDER CANCER

**Chemotherapy**

Cisplatin-based combination chemotherapy (i.e., ddMVAC and GC) has long been the standard of care in metastatic bladder cancer, demonstrating an OS in the range of 9 to 15 months (Kantarjian & Wolff, 2016; von der Maase et al., 2005). Long-term survival benefits are similar between MVAC and GC, although GC has been found to be less toxic and has therefore emerged as the standard of care (von der Maase et al., 2005). Just as in the neoadjuvant setting, GTA has shown activity in the metastatic setting for patients with altered renal function (Siefker-Radtke et al., 2016). Prior to the approval of atezolizumab in 2016, there was no standard of care for second-line therapy in the metastatic setting (Campbell et al., 2016).

**Checkpoint Inhibitors**

Programmed cell death ligand 1 (PD-L1), programmed cell death protein 1 (PD-1), and cytotoxic T-lymphocyte–associated antigen 4 (CTLA-4) blockade have emerged in the past decade, representing a paradigm shift in cancer care (Campbell et al., 2016). Immune checkpoint inhibition has yielded significant survival benefits in a number of malignancies, including melanoma, non–small cell lung cancer, renal cell carcinoma, head and neck malignancies, and urothelial carcinomas (Rosenberg et al., 2016). Rosenberg and colleagues (2016) completed a single-arm, multicenter, phase II trial which included 310 patients with metastatic urothelial carcinoma who had progressed after platinum-based chemotherapy; results showed an overall response rate of 15% with atezolizumab (Tecentriq), an improvement from the 10% seen in historical controls (*p* = .0058). The approval of atezolizumab in 2016 marked the first breakthrough in metastatic bladder cancer therapy since MVAC was found to have significant activity in 1985.

Since atezolizumab’s initial approval, the market has been flooded with four additional approvals of checkpoint blockade, including durvalumab (Imfinzi) and avelumab (Bavencio; PD-L1 blockade) as well as nivolumab (Opdivo) and pembrolizumab (Keytruda; PD-1 blockade; NCCN, 2018). All five agents are approved in the second-line setting following progression of metastatic urothelial carcinoma on previous platinum-based chemotherapy, with pembrolizumab and atezolizumab holding additional approvals in the front-line setting in those patients deemed platinum ineligible (NCCN, 2018).

With five new approvals of relatively similar immunotherapies, it can be challenging to know which agent is best for patients with metastatic urothelial carcinoma. Of the five available agents, pembrolizumab is currently the only checkpoint inhibitor in urothelial carcinoma with category 1 evidence from a phase III trial showing improved OS benefit in the postplatinum setting (Bellmunt et al., 2017). The pivotal KEYNOTE-045 study, which compared pembrolizumab to investigator’s choice of chemotherapy in metastatic urothelial carcinoma, reported an objective response rate of 21.1% as compared to 11% in the chemotherapy arm (Bellmunt et al., 2017). The pembrolizumab arm had a median OS of 10.3 months, as compared to 7.4 months in the chemotherapy arm; this survival benefit was maintained at 18.5 months regardless of PD-L1 expression, investigator’s choice of chemotherapy, histology, prior therapy, age, or performance status (Bellmunt et al., 2017). Furthermore, the every-3-week dosing schedule makes this a convenient option for patients.

As with other immune checkpoint inhibitors, toxicities are primarily immune-mediated; careful monitoring for pneumonitis, colitis, hepatitis, hypophysitis, and dermatitis is essential. There are several ongoing clinical trials with immune checkpoint inhibitors in urothelial cancer; further areas of exploration include combinations of immunotherapy and chemotherapy, optimal treatment sequencing, and identifying resistance mechanisms (McConkey et al., 2015). The future of advanced urothelial carcinoma is at last showing promising signs of progress.

## URACHAL CARCINOMA

Urachal carcinoma is a rare entity, accounting for 0.35% to 0.7% of all bladder cancers (Gopalan et al., 2009). These tumors are typically histologically adenocarcinomas, arising from the urachas, a remnant from embryonic development, or the dome of the bladder (Gopalan et al., 2009; Siefker-Radtke et al., 2003; Szarvas et al., 2016). There is currently no standard of care for the management of urachal tumors; however, there are case reports and retrospective studies to help guide treatment decisions.

Management of nonmetastatic urachal tumors is typically surgical; both partial and complete cystectomies yield similar results (Szarvas et al., 2016). A complete en bloc resection, where the urachal remnant and the umbilicus are completely removed, yields the greatest prolonged survival (Szarvas et al., 2016). In a retrospective review by Siefker-Radtke and colleagues (2009), the majority of survivors (81%) had a complete en bloc resection at the time of surgery, further supporting its significance.

There is currently no standard for systemic therapy in the metastatic setting, although cisplatin and 5-FU combination therapies have yielded the most favorable data and are commonly used (Szarvas et al., 2016). The combination of 5-FU, leucovorin, gemcitabine, and cisplatin (Gem-FLP) has shown promising results; Siefker-Radtke and colleagues (2003) reported an objective response rate of 33% using this regimen. This regimen can also be considered in urothelial carcinomas with pure adenocarcinoma histology. Further studies are needed to standardize treatment for this tumor type; however, this can prove challenging in such a rare tumor.

## DISCUSSION

Bladder cancer remains a challenging disease for clinicians and patients alike, with curative intent requiring vigorous chemotherapy regimens and close monitoring. Furthermore, the 5-year relative survival rate for stage 2 bladder cancer is 63% and 46% for stage 3 disease—there is clearly room for improvement (American Cancer Society, 2016). Currently, it is standard practice that all patients with MIBC receive NAC; however, there is a subset of patients that might be able to safely avoid NAC; molecular subtyping could potentially identify these patients (Kamat et al., 2016; McConkey et al., 2015). The addition of immune checkpoint inhibition in the metastatic setting as well as an increased understanding of the biology of bladder cancer is finally beginning to change the landscape of this disease.

**Future Considerations**

Muscle-invasive bladder cancers are a heterogeneous group of tumors demonstrating inconsistent response to therapy. Currently, there is an effort underway to further characterize these tumors to better select candidates who will benefit from NAC. At present, it is estimated that 5% to 15% of patients yield survival benefits from NAC, so being able to identify the tumor subtypes of this cohort has obvious treatment implications (McConkey et al., 2015). One readily available tool is molecular profiling; many academic centers have biomarker panels that are utilized to identify mutations. However, the utility beyond trial eligibility remains unclear and improved classification systems are needed. Pulling from the breast cancer data, where intrinsic subtypes are well understood and play a role in treatment decisions, there is hope that these same strategies can be identified and employed in the bladder disease space (McConkey et al., 2015).

McConkey and colleagues (2015) investigated different intrinsic subtypes of MIBCs and their characteristics. Initial pattern observations characterize basal and luminal subtypes where basal MIBCs are noted to be more aggressive, are often metastatic at presentation, and more chemosensitive and immunosensitive. Luminal MIBCs tend to have more papillary histopathologic features, are more common in micropapillary MIBC, and often have *FGFR3* mutations (McConkey et al., 2015). This suggests that luminal MIBCs are initially superficial cancers that have progressed to muscle invasion (Kamat et al., 2016; McConkey et al., 2015). It is still too early to extrapolate a practice change based on these findings; however, further research should continue to investigate these associations for clinically relevant applications in the bladder cancer setting.

**Implications for Advanced Practice Providers**

Multidisciplinary care continues to be emphasized in healthcare, and oncology is no exception. Advanced practice providers (APP) play a key role in ensuring the development and execution of treatment plans, and therefore must possess sound fundamental knowledge of treatment regimens, toxicity profiles, and treatment goals. Bladder cancer is the sixth most common malignancy; therefore, oncology APPs will encounter patients with bladder cancer and need to be well versed in the management (American Cancer Society, 2016). Many of the above-mentioned treatment regimens require close monitoring and follow-up; APPs possess the ideal set of skills to carry out the appropriate monitoring, patient education, and symptom management while patients are under active treatment.

## CONCLUSION

Bladder cancer is a common malignancy that necessitates aggressive management and coordination between multidisciplinary teams. This review provides an overview of the currently approved treatment strategies for advanced bladder cancer. Despite a prolonged period of stagnation in new treatment strategies, the future looks promising. The addition of immune checkpoint inhibitors in the metastatic setting has marked a new era for bladder cancer. Further research using checkpoint inhibitors has yielded promising results and additional approvals of these agents is anticipated. Further optimism has centered on personalized medicine, where agents target specific molecular mutations, such as *FGFR3* and *MTAP*. There is new energy centered on urothelial cancer, and all those involved in the care of these malignancies should feel hopeful about the future of patient outcomes.
